# Machine learning based approach to pH imaging and classification of single cancer cells

**DOI:** 10.1063/5.0031615

**Published:** 2021-03-16

**Authors:** Y. Belotti, D. S. Jokhun, J. S. Ponnambalam, V. L. M. Valerio, C. T. Lim

**Affiliations:** 1 Institute for Health Innovation and Technology, National University of Singapore, 117599 Singapore, Singapore; 2 Department of Biomedical Engineering, National University of Singapore, 117583 Singapore, Singapore; 3 Mechanobiology Institute, National University of Singapore, 117411 Singapore, Singapore

## Abstract

The ability to identify different cell populations in a noninvasive manner and without the use of fluorescence labeling remains an important goal in biomedical research. Various techniques have been developed over the last decade, which mainly rely on fluorescent probes or nanoparticles. On the other hand, their applications to single-cell studies have been limited by the lengthy preparation and labeling protocols, as well as issues relating to reproducibility and sensitivity. Furthermore, some of these techniques require the cells to be fixed. Interestingly, it has been shown that different cell types exhibit a unique intracellular environment characterized by specific acidity conditions as a consequence of their distinct functions and metabolism. Here, we leverage a recently developed pH imaging modality and machine learning-based single-cell segmentation and classification to identify different cancer cell lines based on their characteristic intracellular pH. This simple method opens up the potential to perform rapid noninvasive identification of living cancer cells for early cancer diagnosis and further downstream analyses.

## INTRODUCTION

For many biological and biomedical applications, immunofluorescence has been widely used over the last few decades to visualize specific biological phenomena occurring at the cellular and subcellular levels even though it has multiple drawbacks. Firstly, fluorophores can induce phototoxic effects, which are primarily associated with the generation of reactive oxygen species that have been shown to have adverse effects on cell physiology and health.^1^ Although phototoxic damage can be quantified and minimized, it cannot be eliminated.^2^ Moreover, as antibodies are unable to move across the cell membrane, immunofluorescence requires a cell fixation step.^3^ This renders it impossible to perform any further downstream analysis that requires the cells to be alive. Furthermore, research areas, such as *in vitro* stem cell and drug discovery studies, require minimal cell manipulation.^4^ Therefore, new efficient and sensitive alternative methods are needed to enable scientists to extract valuable information out of living cells. Additionally, to account for the inherent heterogeneity associated with biological samples, single-cell information is often required.

Among other approaches, looking at intracellular acidity has been shown to be a valuable option to study single cells. Specifically, intracellular acidity is directly associated with many physiological processes, such as cell migration,^5,6^ division,^7^ and apoptosis,^8,9^ and affects how the whole cellular environment functions by controlling events spanning from enzymatic activity to cytoskeletal structure dynamics.^10–12^ Physiological pH varies between 4.7 and 8.0,^13,14^ and deviations from healthy intracellular acidity have been linked with the onset of various diseases such as Alzheimer's and even heat stroke.^15,16^ Furthermore, cancer growth, invasion, and metastasis have been associated with abnormal levels of cytosolic pH.^17,18^ The roles of dysregulated pH dynamics in cancer initiation, progression, and adaptation have been recently highlighted by White and colleagues.^19^ Specifically, in cancer cells, the intracellular pH tends to be higher than in normal cells, whereas the extracellular pH follows the opposite trend. This phenomenon has been observed in the early phases of cancer development,^20^ and the differences in pH between the intracellular and extracellular environment tend to increase during neoplastic progression.^21^ Increased intracellular pH has been proposed to be associated with epithelial-to-mesenchymal transition,^22^ which is linked with metastatic initiation. Various methods have been developed to study cellular pH, mainly relying on fluorescence indicators^23–26^ and decorated nanoparticles.^17,27,28^ However, they have limitations such as complex multi-step protocols for synthesis and functionalization of nanoparticles. Moreover, fluorescence imaging methods are commonly affected by photobleaching, which is known to affect cell physiology.^1^ In 2017, Hou *et al.* reported for the first time a novel single-cell pH-based imaging method, where the authors were able to rapidly identify cancer cells by combining UV-vis micro-spectroscopy and the use of common pH indicators.^29^

Numerous advancements in the field of computer vision enabled innovative approaches to extract valuable information from biological and medical images.^30–32^ Specifically, various Machine Learning (ML) based algorithms have been developed to obtain multiple features from single cells and even subcellular components and used to identify complex phenotypes and diagnose diseases.^33,34^

Here, we report a novel approach that combines quantitative pH-based colorimetric imaging with ML-based single-cell segmentation and classification. Using this method, we aimed to differentiate nontumorigenic from cancerous breast cells purely on their intracellular acidity conditions. Furthermore, we sought to extend the analysis to the classification of human single cells of various tissues, both normal and cancerous.

## RESULTS

### Single-cell pH-based colorimetric imaging

The first step of our study was to develop and optimize a facile colorimetric imaging approach that would allow us to differentiate among various cell lines of the same or different organs, based on characteristic intracellular pH levels. Specifically, we sought to test whether we could successfully classify two breast cell lines: MCF-10A and MDA-MB-23. Next, we included in our study the pancreatic cancer cell line Mia-PaCa-2 and the human umbilical vein endothelial cells (HUVECs). To implement a pH-based imaging modality, the pH-sensitive dye Bromothymol Blue (BTB) was used. BTB needs to be internalized by the cells, as Hou *et al.*^29^ previously demonstrated by incubating cells with aqueous ethanol solution. The authors showed that this treatment increases the permeability of the cells to BTB. We first determined the highest ethanol concentration that could be tolerated by the various cell lines under investigation. Moreover, the incubation time was additionally tested. Figure S1 shows the results of the viability tests conducted by varying the concentration and the incubation time. While incubating for only 5 min at ethanol (EtOH) concentration of 20% caused a dramatic drop in cell viability, the cell viability was ∼98.9% for lower EtOH concentrations, for all the cell lines under investigation. Given the similarity in viability profiles between MCF-10A, MDA-MB-231 and HUVEC, we then sought to test the viability of MDA-MB-231 and MiaPaCa-2 by incubating them for 15 and 30 min, at increasing concentrations of EtOH (0.1%, 0.5%, 1%, 5%, and 10%). For both cell lines, the viability was high at all the tested concentrations and incubation times, and hence, we decided to use the EtOH concentration of 5% and an incubation time of 15 min for the BTB internalization part of our protocol. To further investigate the effect of ethanol treatment, we assessed cell growth over time. After incubating cells with 5% EtOH for 15 min, cells were washed once with Dulbecco's Phosphate Buffered Saline (DPBS) and culture media were added. MDA-MB-231 (passage number 10) was monitored for 72 h as it has the longest doubling time (38 h). MCF-10A (passage number 13), MiaPaCA-2 (passage number 20), and HUVECS (passage number 8) have doubling times of 16, 22, and 23 h, respectively, and as such, we followed their growth for 48 h. The growth curves are shown in Fig. S2. We decided not to extend our analysis beyond 72 h for MCF-10A and 48 h for the other cell lines as any change in the growth curves beyond these time points would be relative to cells that belong to successive generations, which did not undergo ethanol treatment.

**FIG. 1.**
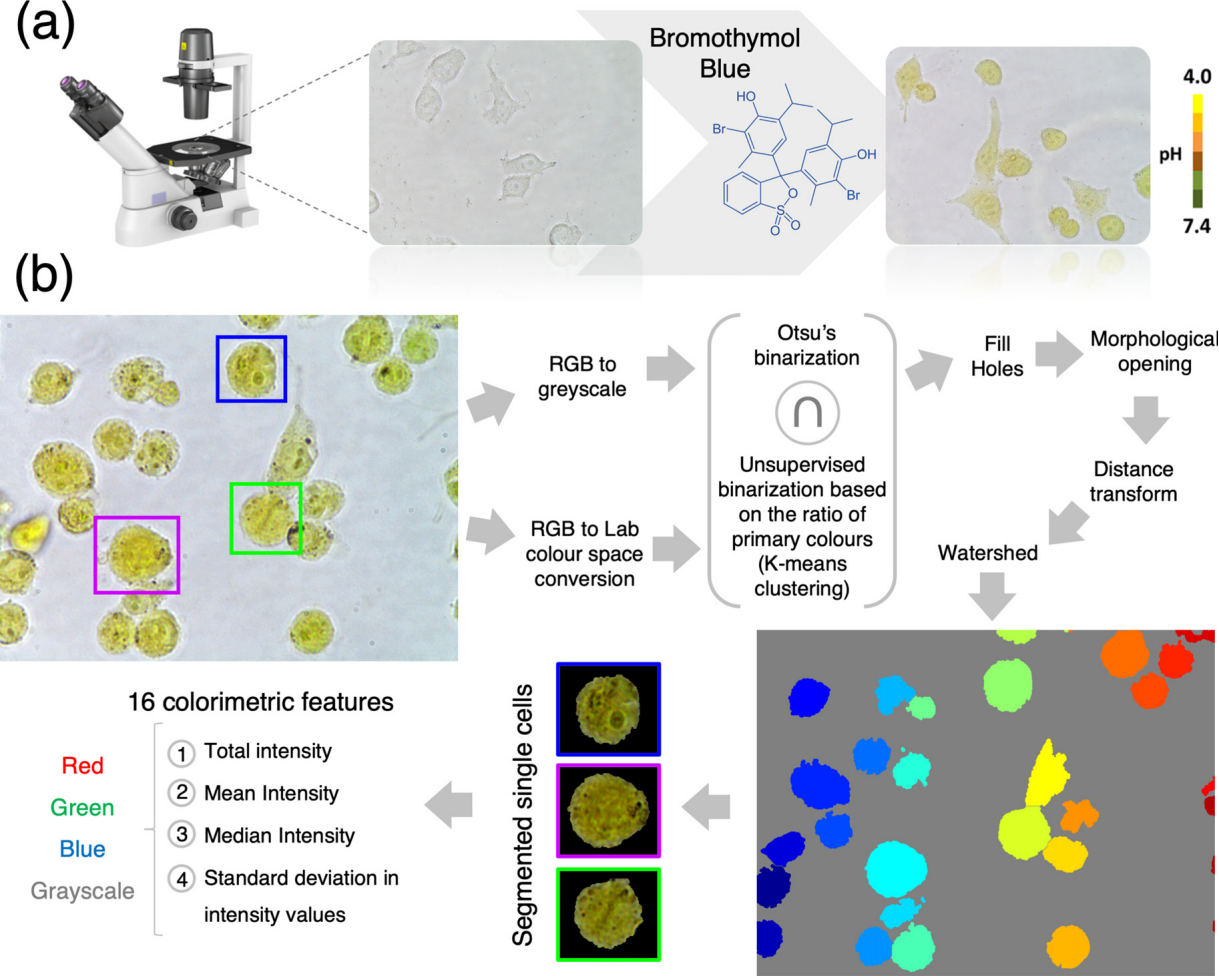
Single-cell pH-based imaging, segmentation, and feature extraction workflow. (a) Workflow of the colorimetric pH imaging. Color images are acquired using an optical microscope equipped with a color digital camera. (b) Automated single-cell segmentation pipeline. Raw images are converted to the L*a*b color space and each pixel classified as being a “background pixel” or “cell pixel” using k-means clustering. In parallel, RGB images are converted to the grayscale and thresholded using Otsu's method. A mask is created by selecting only the pixels that were classified as cell pixel by both methods simultaneously. Watershed is used to segment individual cells. Finally, 16 colorimetric features are expected from each cell.

**FIG. 2.**
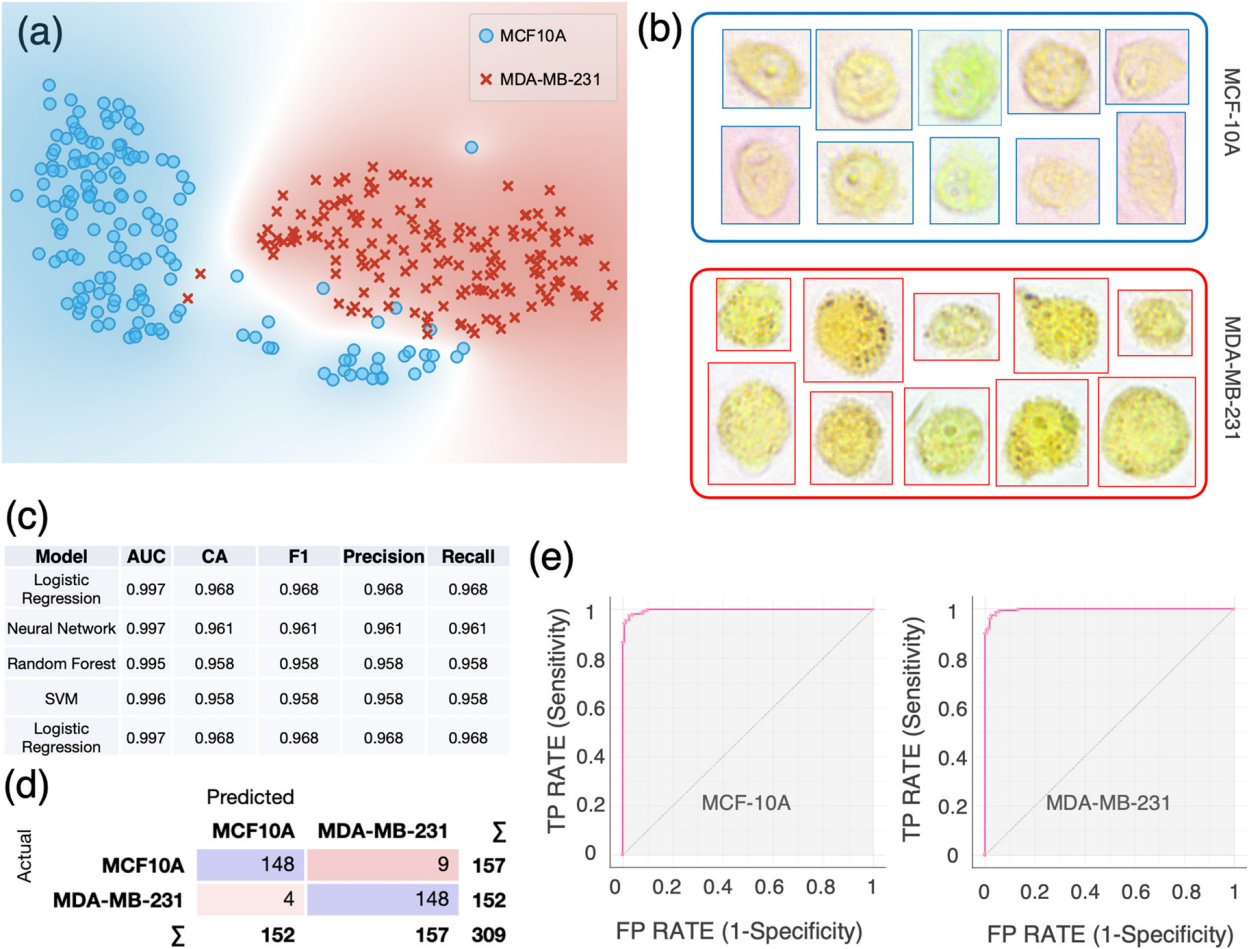
Single-cell computational classification of breast cell lines: MCF-10A and MDA-MB-231. (a) The t-SNE plot of two breast cell lines. (b) Examples of color bright-field images of individual MCF-10A and MDA-MB-231 cells. Cells are imaged just after incubation with BTB using a 40× objective. (c) Evaluation results of the models using 10-fold cross-validation. AUC = Area Under the Curve; CA = classification accuracy. The table is sorted based on the scoring values of CA and precision. (d) Confusion matrix of the logistic regression model, which best scored in the classification. (e) Receiver operating characteristic (ROC) curves for each cell line using logistic regression. The size of each sample is N = 157 for MCF-10A and N = 152 for MDA-MB-231.

Next, we aimed to determine the working concentration of BTB by investigating the effects of the BTB on cell viability by testing three different concentrations of 0.25 mg/ml, 0.5 mg/ml, and 1 mg/ml. The internalization increased by increasing the BTB concentration, as shown in Fig. S3, in which the intensity exhibited by MiaPaCa-2 cells just after incubation with BTB at increasing concentrations is shown. Finally, the long-term effects associated with the increasing concentration of BTB were tested [Fig. S3(b)] after 2 days. At concentrations of 0.25 and 0.5 mg/ml, cells appear mostly viable and actively spreading, similar to the control case. On the other hand, at 1 mg/ml, which is the concentration used in the previous study by Hou *et al.*,^29^ most of the cells are rounded, suggesting possible cytotoxic effects. Taken together, our results indicate that the best trade-off between internalization and viability is the intermediate concentration of 0.5 mg/ml, which was then adopted in our protocol.

**FIG. 3.**
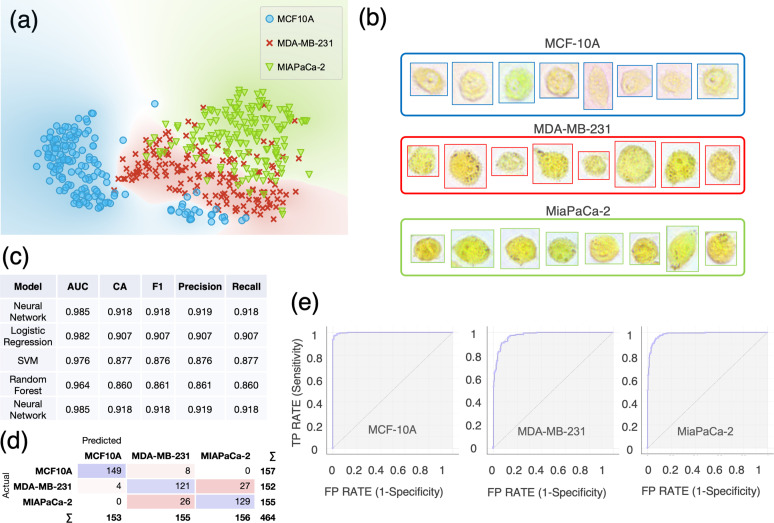
Single-cell computational classification of three cell lines: MCF-10A, MDA-MB-231, and MiaPaCa-2. (a) The t-SNE plot of three cell lines. (b) Examples of color bright-field images of individual MCF-10A, MDA-MB-231, and MiaPaCa-2 cells. Cells are imaged just after incubation with BTB using a 40× objective. (c) Evaluation results of the modeling using 10-fold cross-validation. AUC = Area Under the Curve; CA = classification accuracy. The table is sorted based on the scoring values of CA and precision. (d) Confusion matrix of the neural network model, which best scored in the classification. (e) Receiver operating characteristic ROC curves for each cell line using the neural network model. The size of each sample is N = 157 for MCF-10A, N = 152 for MDA-MB-231, and N = 155 for MiaPaCa-2.

### Imaging, automated single-cell segmentation, and feature extraction

Each experiment started by seeding cells in three separate wells of a standard 24-well plate to allow for technical replicates. In each well, culture media were removed and replaced with 5% ethanol solution (in DPBS) and incubated for 15 min in an incubator. The ethanol solution was then replaced with 0.5 mg/ml BTB of solution (in DPBS) and incubated for 15 min in an incubator. Finally, the BTB solutions were removed and the wells were washed three times with DPBS to remove excess BTB. Bright-field images were subsequently acquired using an inverted microscope (Nikon Eclipse TS100) equipped with a digital color camera (Nikon Digital Sight DS-Fi1c) [Fig. 1(a)]. The shape and position of each cell in the raw color [Red/Green/Blue (RGB)] images were estimated by the simultaneous application of two independent methods (Otsu's and k-means clustering), as shown in Fig. 1(b). Finally, watershed was used to segment individual cells and colorimetric features were extracted and exported into a spreadsheet file. The workflow of our approach is summarized in Fig. 1. Examples of the output of our segmentation can be seen in Fig. S4.

**FIG. 4.**
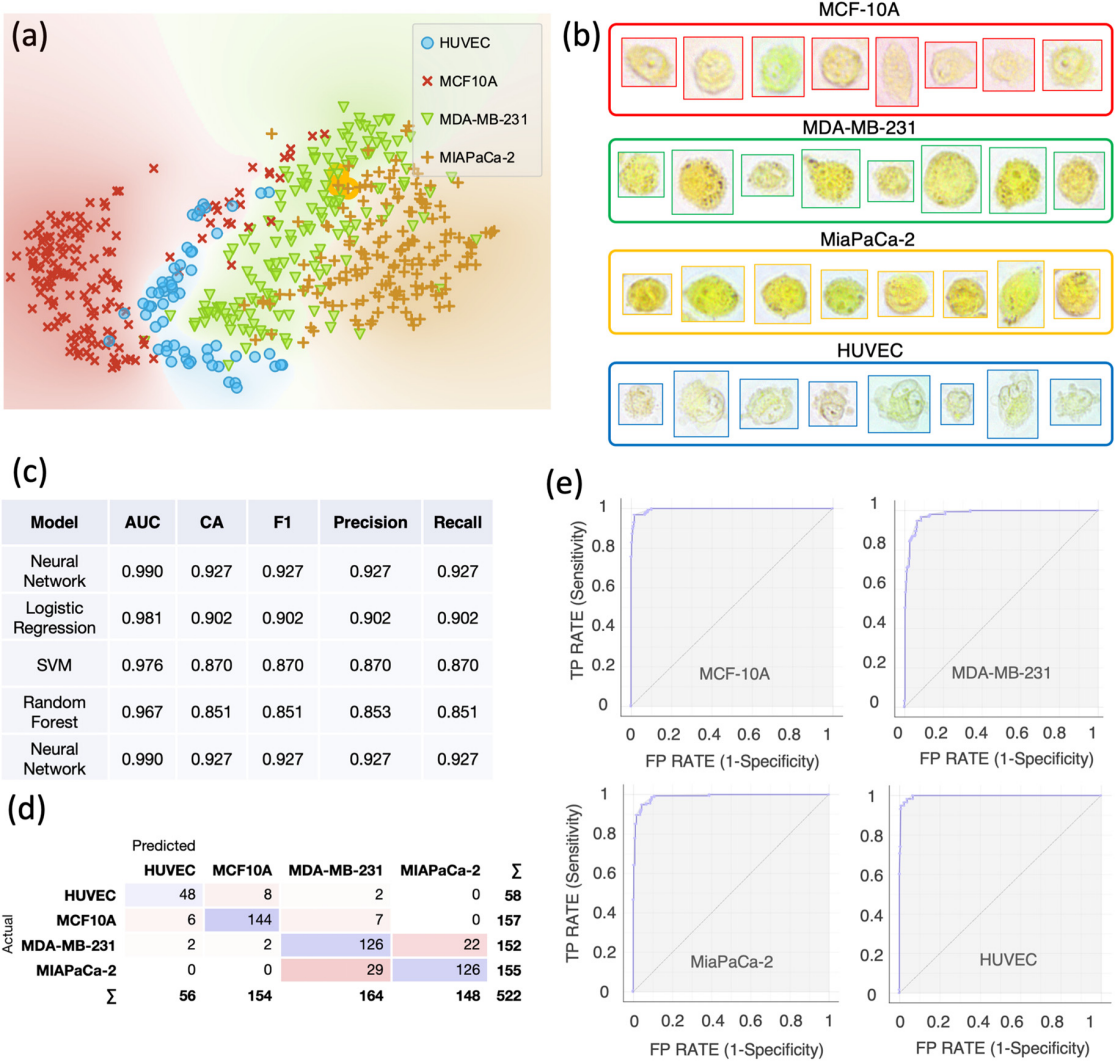
Single-cell computational classification of four cell lines: MCF-10A, MDA-MB-231, and MiaPaCa-2 and HUVEC. (a) The t-SNE plot of four cell lines. (b) Examples of color bright-field images of individual MCF-10A, MDA-MB-231, MiaPaCa-2, and HUVEC cells. Cells are imaged just after incubation with BTB using a 40× objective. (c) Evaluation results of the modeling using 10-fold cross-validation. AUC = Area Under the Curve; CA = classification accuracy. The table is sorted based on scoring values of CA and precision. (d) Confusion matrix of the neural network model, which best scored in the classification. (e) Receiver operating characteristic ROC curves for each cell line using the neural network model. The size of each sample is N = 157 for MCF-10A, N = 152 for MDA-MB-231, N = 155 for MiaPaCa-2, and N = 58 for HUVECs.

### Single-cell computational classification: Noncancerous vs cancerous breast cells

A single-cell classification “visual algorithm” was developed in Orange (the workflow shown in Fig. S5). We first sought to apply our single-cell classification method to two breast cell lines: MCF-10A, a human nontumorigenic mammary epithelial cell line, and MDA-MB-231, a known metastatic breast cancer cell line. Datasheets containing the single-cell colorimetric features were imported in Orange for further data analysis and visualization. The t-distributed Stochastic Neighbor Embedding (t-SNE) plot in Fig. 2(a) shows a neat separation between the two cell lines. These two cell lines exhibited a characteristic color profile, as shown in Fig. 2(b). To make sure that no correlation was present among the features used for the cell classification, we conducted a principal component analysis (PCA) and the first six PCs were selected to achieve 99% explained variance. Multiple supervised learners were then applied to the transformed data and their performances compared. The best classification results between nontumorigenic and metastatic breast cells were obtained using a logistic regression classifier: the accuracy and precision of 96.8% [Fig. 2(c)] were achieved using a 10-fold cross-validation for model testing. For both cell lines, the diagnostic ability of the binary classifier system is illustrated by the confusion matrix in Fig. 2(d) and the receiver operating characteristic (ROC) curves in Fig. 2(i). Further validation using an “*in silico* co-culture” as validation dataset resulted in an accuracy and precision of 95.1% and 95.3% using a Support Vector Machine (SVM) model [Fig. S7(a)].

**FIG. 5.**
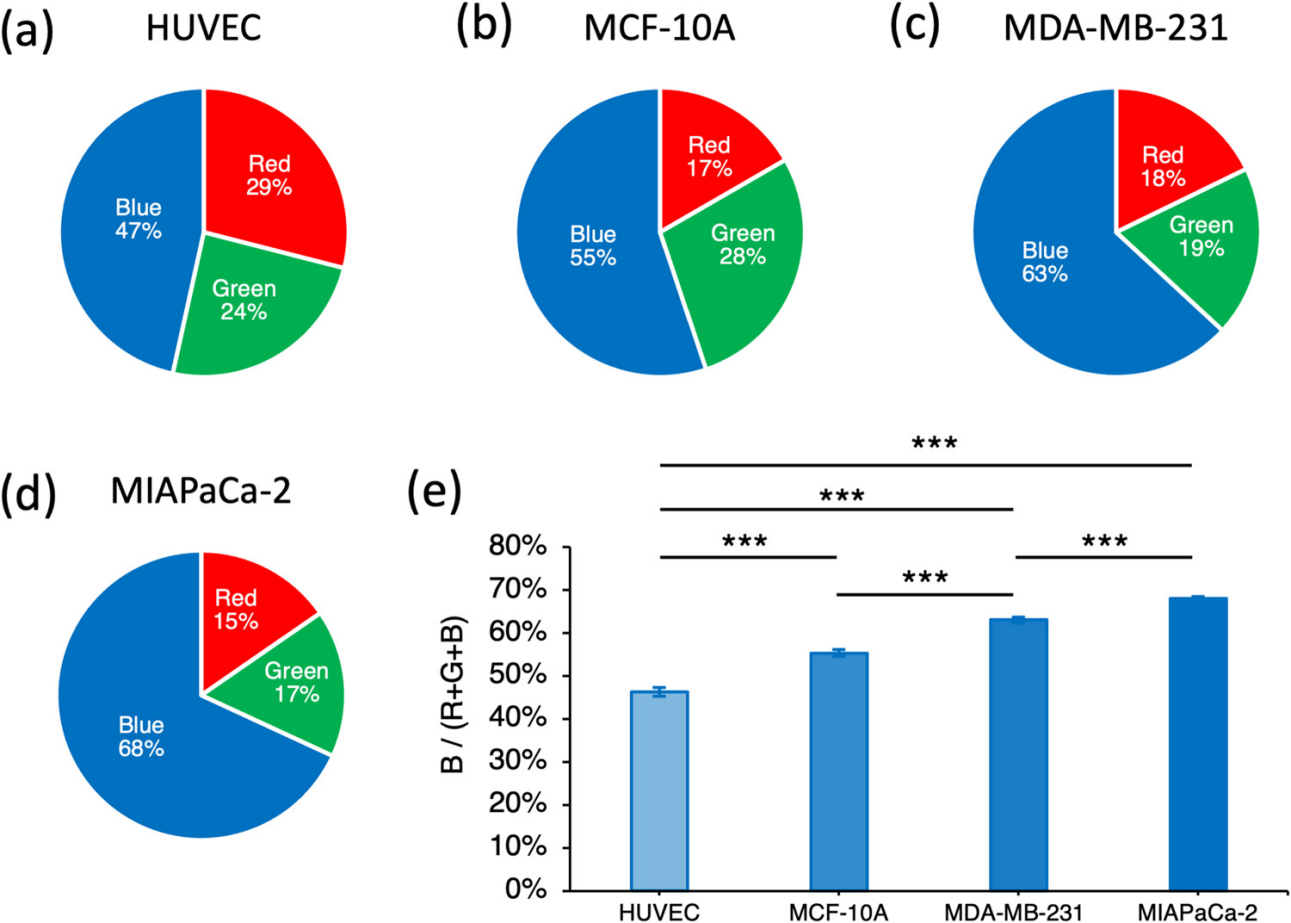
RGB analysis. The pie charts show the Red/Green/Blue components of the single-cell images for the following cell lines: (a) HUVEC, (b) MCF-10A, (c) MDA-MB-231, and (d) MiaPaCa-2. (e) Quantification of the blue signal with respect to the total intensity signal (Red + Green + Blue) for each cell lines. The histogram shows the average of the median intensity values of the blue signal calculated across each cell. Error bars represent 95% confidence interval. Paired samples t-tests are performed. ***p < 0.001.

### Single-cell computational classification: Multiple cell lines

Moreover, we sought to test whether we could successfully differentiate the cell lines previously analyzed from an additional one belonging to a different organ. For this, we used the human pancreatic cancer cell line Mia-PaCa-2. This third cell line exhibited a distinguishable, characteristic color profile compared with the other two previously analyzed [as shown in Fig. 3(b)]. Even in the presence of three different cell types, the t-SNE plot [Fig. 3(a)] shows a neat separation among the three cell populations. In this case, the MCF-10A ROC curve exhibits an almost ideal profile [in Fig. 3(i)], whereas the other two ROC curves indicate that the classification occurred with slightly lower sensitivity and specificity compared to MCF-10A. In this case, a neural network model best scored in the single-cell classification achieving 91.8% accuracy and 91.9% precision using 10-fold cross-validation for model testing, as shown in Fig. 3(c). Further validation using an *in silico* co-culture dataset resulted in an accuracy and precision of 85% and 86% using an SVM model [Fig. S7(b)].

Finally, we sought to extend our classification to a cell line from a normal human tissue: human umbilical vein endothelial cells (HUVECs). Once again, a neat separation among the groups can be seen in the t-SNE plot [Fig. 4(a)] and all the cell lines exhibited a different color profile [Fig. 4(b)]. Both the classification accuracy (CA) and precision were 92.7%, as shown in Fig. 4(d). Further validation using an *in silico* co-culture dataset resulted in an accuracy and precision of 78% and 79.9% using a neural network model [Fig. S7(c)].

Characteristic RGB ratios were exhibited by each cell line, as shown in Figs. 5(a)–5(d). Moreover, the blue signal, which is associated with the color change of the BTB, was significantly lower (*p *<0.001) in HUVECs if compared with all the other cell lines [Fig. 5(i)]. Significantly higher pH levels (*p *<0.001) were observed in MDA-MB-231 with respect to MCF-10A. The highest pH (*p *<0.001) levels were measured in MiaPaCa-2. We also sought to understand whether our method could work in the absence of BTB treatment, by leveraging the potential inherent colorimetric differences between cell lines. Figure S6(a) shows the outcomes of the classification between MDA-MB-231 and MiaPaCa-2 where images were acquired without BTB. A classification accuracy of 87% was achieved. The same cell lines were also imaged using BTB and a classification accuracy of 91% was achieved [Fig. S6(b)], indicating that the presence of the BTB enhanced the classification performance. Furthermore, we sought to assess the importance of acquiring RGB compared to grayscale features only. We then performed a classification of the four cell lines shown in Fig. 4, but in the first case, we used only grayscale-related features, whereas in the second case, we used RGB-related features only. The results are summarized in Fig. S8, where it is visible that the use of RGB-related features leads to superior classification performances.

**FIG. 6.**
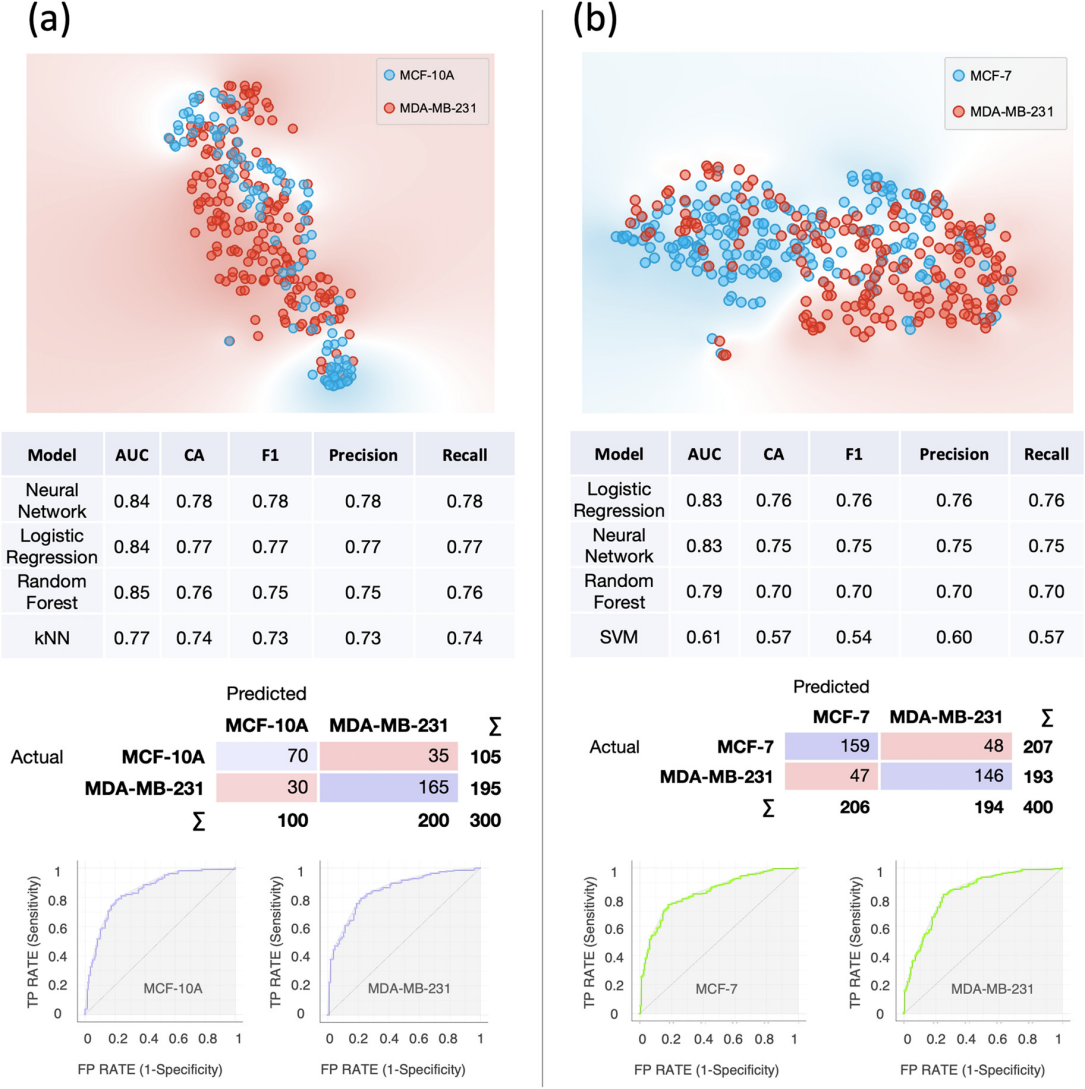
Single-cell computational classification in the case of co-cultures. (a) Results of the computational classification between MCF-10A (N = 136) and MDA-MB-231 (N = 164). (b) Results of the computational classification between MCF-7 (N = 117) and MDA-MB-231 (N = 283). In both cases, at the top, the t-SNE plots are shown; in the middle, the evaluation results of the modeling using 10-fold cross-validation and confusion matrices for the best scoring models are shown. AUC = Area Under the Curve; CA = classification accuracy. Below, the ROC curves for each cell line relative to the best scoring models are shown.

### Single-cell computational classification in co-cultures

To further validate our method, we applied it to images acquired from co-cultures. Specifically, we co-cultured MDA-MB-231 with MCF-10A and MDA-MB-231 with MCF-7. In the first case, we sought to extend our first analysis (shown in Fig. 2) by co-culturing the two cell lines. In the second case, we sought to investigate whether we could differentiate two physiologically similar breast cancer cell lines, as they are both derived from metastatic sites. An accuracy of 78% was achieved in the first case, whereas an accuracy of 76% in the second case, as shown in Fig. 6.

## DISCUSSION

In this study, we developed a novel protocol for noninvasive, single-cell classification based on the characteristic values of intracellular pH exhibited by different cell lines. Our protocol combines bright-field color imaging and automated ML-based image analysis. The pH-imaging principle is based on a recent work by Hou *et al.*^29^ where for the first time, live-cell imaging and single-cell intracellular pH sensing and profiling were achieved and used for cancer cell identification. In our study, we conducted a more in-depth analysis to identify the range of ethanol concentrations to enable dye internalization without affecting the cellular physiology. We then tested the short- and long-term effects of the exposure to the pH-sensitive dye Bromothymol Blue at different concentrations and found that the concentration of 1 mg/ml, previously reported by Hou *et al.,*^29^ was not well tolerated by all the cells included in our investigation. Hence, our results suggested that lower BTB concentrations should be used. Specifically, we found that the best trade-off in terms of BTB internalization and cell viability was 0.5 mg/ml. We then developed an *ad hoc* computational tool to automatically extract single-cell optical information associated with the characteristic color profiles acquired by each cell after the incubation with BTB. Furthermore, we leveraged the power of Orange, open-source visual programming software for data mining and ML, to classify single cells based on the specific colorimetric features associated with their intracellular pH levels. Using our segmentation algorithm, images of single cells were automatically segmented and colorimetric features were extracted from the raw images. Using our single-cell classification algorithm, we found that various cell lines could be classified based on their intracellular pH with an accuracy >90%, in all the analyzed cases. Specifically, we were able to differentiate between nontumorigenic and metastatic breast cancer cells and among human cell lines from various tissues, both normal and cancerous. Our method was further validated using “*in silico* co-cultures” datasets and in actual co-cultures, where a classification accuracy of ∼78% was achieved in the case of MCF-10A and MDA-MB-231 and ∼77% in the case of two cell lines that are expected to exhibit similar pH values as they are both metastatic breast cancer cell lines from the same site (MCF-7 and MDA-MB-231). We also demonstrated the potential use of our method in the absence of BTB internalization, by leveraging the inherent colorimetric differences between cell lines. Although results from Fig. S6 show that BTB enabled better computational classification, being able to classify cells without using BTB looks promising too and will enable development toward a label-free version of our approach.

Interestingly, lower levels of pH were found in normal cells (HUVECs) when compared with all the cancer cell lines. Moreover, higher pH levels were measured in the metastatic breast cancer cell line MDA-MB-231 when compared with the noncarcinogenic MCF-10A. These results are in line with the previous literature.^20,21^ Increased intracellular pH in cancer cells is maintained by the enhanced activity of plasma membrane ion transporters and pH regulators such as Na^+^-driven HCO_3_ exchangers,^35^ Na^+^–H^+^ exchangers 1 (NHE1),^36^ monocarboxylate transporter 1 and 4 (MCT1 and MCT4),^37^ and carbonic anhydrases (Cas).^38^

Many studies involving pH-based sensors require cells to internalize nanoparticles, which tend to remain in the lysosome by endocytosis unless modified with specific targeting ligands.^39^ Our method relies on a dye internalization step that increases the permeability of the cell membrane allowing the BTB to homogenously diffuse across it.

Other noninvasive imaging modalities have been developed over the last decade such as digital holographic microscopy (DHM),^40–42^ transport-of-intensity equation (TIE) based imaging,^43^ and ptychography.^4,44,45^ They are all aimed at overcoming the limitations of genetic and synthetic fluorescent labels. However, the implementation of these imaging techniques requires rather complex and expensive optical systems. Novel high-throughput technologies recently emerged to identify and classify different cell types and the effects of specific drugs based on intrinsic cellular properties of single cells in suspension.^46–48^ Although allowing for minimal cell manipulation and being valuable diagnostic methods, on their own or in combination with other systems for multiplexed analyses,^49^ these methods require high-end, high-speed imaging systems to acquire images of single cells flowing at high flow rates through microfluidic devices. Further developments of our method are warranted to extend it to cell suspensions, potentially increasing its throughput, and integrating it in lab-on-a-chip applications, flow cytometry, and cell sorting.

Our approach can also be used for subcellular pH detection. Different cellular compartments are characterized by different pH levels, which have been previously reported.^29^ For instance, it has been shown that some organelles, such as the nucleus, endoplasmic reticulum, and peroxisomes, lack intrinsic pH-regulatory systems but seem to readily equilibrate their luminal pH to the cytoplasmic levels.^50^ Based on our observations, this might differ based on the organ of origin of the cells analyzed. In fact, in some cases, BTB internalization within different organelles appeared with high contrast compared with the cytosolic environment [e.g., Fig. 2(b), MCF-10A, and MDA-MB-231], while for other cell lines, the spatial color distribution appeared more homogeneous [e.g., Fig. 3(b), MiaPaCa-2].

Our study presents a few limitations. Firstly, we restricted our investigation to four cell lines. Therefore, the effects of EtOH and BTB on other cell types remain to be explored. Secondly, the only primary cells included in our study were the HUVECs. Hence, the extension to other primary cells is warranted. Thirdly, a direct comparison with conventional fluorescent pH indicators was not conducted. Nevertheless, our segmentation and classification pipelines could also be adapted to analyze fluorescence images. Additionally, it has been shown that intracellular pH can be affected by extracellular pH.^51^ To account for this, culture media were supplemented with HEPES [4-(2-hydroxyethyl)-1-piperazineethanesulfonic acid] as pH buffers, and therefore, cell-induced extracellular changes in the pH were constantly compensated. Cells were then pretreated with DPBS and imaging was also conducted in DPBS, which required about 5 min. Furthermore, the number of adherent cells in the 24-well plate was low compared to the volume of DPBS. Therefore, we could argue that pH changes in the media would be negligible within the image acquisition time.

In the future, the extension of our protocol to observe cellular compartments will allow for novel basic and translational studies on intracellular pH distribution and time-dependent analyses in living cells. More studies are required to understand whether this approach could be used to detect cells at different stages of malignancies. The noninvasive nature of our single-cell classification protocol makes it a valuable option for identifying various cell types, and therefore, it could be leveraged for future integration with existing screening and diagnostic methods. At the current stage of development, our protocol requires about 35 min: 15 min for ethanol treatment, 15 min for BTB treatment, 5 min for image acquisition, 10 s for image segmentation, and 10 s for classification. Hence, future development of a real-time version of our approach, where single cells in adherent or suspended conditions can be automatically recognized and individually handled, could represent an inexpensive alternative to standard flow cytometry-based cell sorting. This would have the advantage of maintaining cells viable and, therefore, opening up the potential for downstream analyses in separate or integrated devices.

## CONCLUSION

We developed a novel method for noninvasive, single-cell classification based intracellular pH levels and spatial profile exhibited by different cell lines. Specifically, combining bright-field color imaging and machine learning-based approach we successfully classified: nontumorigenic breast cancer cells (MCF-10A), metastatic breast cancer cells (MDA-MB-231), pancreatic cancer cells (MiaPaCa-2), and human umbilical vein endothelial cells (HUVECs). Our method was further validated on *in silico* co-culture datasets as well as on actual co-cultures. This simple method could potentially be used for rapid noninvasive identification of living cancer cells for early cancer diagnosis and further downstream analyses.

## METHODS

### Materials

Bromothymol blue (Cat. No. 114413–25G) and pure ethyl alcohol (Cat. No. E7023) were obtained from Sigma Aldrich (Singapore). Dulbecco's Modified Eagle medium (DMEM, Cat. No. 12–604F), Dulbecco's Phosphate Buffered Saline (1X) (DPBS, Cat. No. 17–512F), Mammary Epithelial Cell Growth Medium Bulletkit (MEGM, Cat. No. CC-3150), and Penicillin and streptomycin (Pen-strep, Cat. No. 17–602F) were purchased from Lonza (Singapore). Fetal bovine serum (FBS, Heat Inactivated, Cat. No. 10082147), LIVE/DEAD Viability Kit (Cat. No. L3224), and TryPLE Express (1X, Cat. No. 12604013) were purchased from Thermo Fisher Scientific (Singapore). All the chemicals were used as received and without any further purification. All the aqueous solutions were prepared using de-ionized (DI) water, prepared in a Millipore Milli-Q purification system and with a resistivity of 18.2 MΩ cm.

Ethics approval was not required.

### Cell cultures

Four human cell lines were investigated: MiaPaCa-2 (CRL-1420, pancreatic cancer), MCF-10A (CRL-10317, breast epithelial), MDA-MB-231 (HTB-26, mammary gland, breast; derived from metastatic site: pleural effusion), HUVEC (CRL-1730, umbilical vein/vascular endothelium), and MCF-7 (HTB-22, mammary gland, breast; derived from metastatic site: pleural effusion). They were all obtained from ATCC (American Type Culture Collection). All cultures were cultured in a plastic cell culture flask (SPL, Korea) in humidified 37 °C incubators supplemented with 5% CO_2_. All culture flask was maintained in DMEM supplemented with 10% FBS and 1% v/v Pen-strep except for MCF-10A, which was maintained in MEGM. Culture media were replaced every 2 to 3 days. Once samples reached 70% confluency, cells were detached from the flask using 1× TryPLE Express and seeded into 24-well plates at 5 × 10^4^ cells/well. Cultures were allowed to settle for 24-h before experimentation. The same culture media were used for different biological and technical replicates to limit potential variability in their pH.

### LIVE/DEAD cell viability assay

To measure the cytotoxicity of varying concentrations of ethanol *in vitro*, a LIVE/DEAD kit for mammalian cells was used. In brief, 5 × 10^5^ cells/well were seeded in a 24-well plate with their specific culture medium. The cells were allowed to attach for 24 h in a 37 °C, 5% CO_2_ incubator. After incubation, culture medium was removed, 500 *μ*l of varying concentrations of EtOH (0.1%, 0.5%, 1%, 5%, and 10%) was added and incubated for 30 min in a 37 °C, 5% CO_2_ incubator. After 30 min, EtOH was removed and wells were washed two times with DPBS. The cells were then exposed to 500 *μ*l/well LIVE/DEAD assay reagent (2 *μ*l of 2 mM EthD-1 and 0.5 *μ*l of 4 mM calcein AM (acetoxymethyl) in every 1 ml of DPBS) in the dark at RT for 35 min. The reagent was then removed and replaced with 500 *μ*l of DPBS. Calcein AM was viewed using a standard fluorescence bypass filter, while EthD-1 was viewed under Texas red filter. Cells were imaged from three different locations of each well. For each cell line, images were acquired over three biological replicates. Enumeration of LIVE/DEAD cells was conducted in ImageJ using a custom-made macro.

### Cell staining and imaging

Bromothymol blue (BTB) was first dissolved in pure ethanol at the concentration of 20 mg/ml and then diluted in DPBS to the desired concentration (0.25, 0.5, and 1 mg/ml). The cell culture medium was removed from the plates, and adherent cells were incubated with 400 *μ*l of 5% ethanol (v/v in DPBS) for 15 min in the incubator. It was then replaced with 400 *μ*l of BTB solution at the concentration of 0.5 mg/ml (in DPBS) and incubated for 15 min in the incubator. BTB was removed, and the wells washed three times with DPBS to remove noninternalized BTB. Bright-field images were obtained using a Nikon Eclipse TS100 inverted microscope equipped with a digital color camera (Nikon Digital Sight DS-Fi1c). Three technical repeats were acquired by seeding the cells into different wells of a 24-well plate, and three biological replicates were carried out for each cell line.

### Single-cell segmentation and feature extraction

The bright-field images were first converted from the RGB format to the L*a*b color space, and each pixel was classified as being a background or cell pixel according to its L*a*b values using k-means clustering. In parallel, the bright-field RGB images were also converted to the grayscale and thresholded using Otsu's method. A conservative mask was then created by turning on only those pixels that were classified as cell pixel by both methods. Finally, watershed was used on the mask for demarcating and segmenting individual cells. Four classes of intensity features, namely, the total intensity, the mean intensity, the median intensity, and the standard deviation in intensity values were measured at the single-cell level, for each RGB color and grayscale. These were taken from the red, blue, and green components and from the grayscale image of each cell, resulting in a total of sixteen color-based features per cell. These analyses were performed by a custom-written code in MATLAB (MathWorks-Natick, Massachusetts, USA).

### Data visualization

T-distributed Stochastic Neighbor Embedding (t-SNE) plots are used to reduce our high-dimensional data in a two-dimensional map.^52^ In all the t-SNE plots presented, the perplexity is set to 30 and six PCA components are selected to reach the level of 99% explained variance.

### Statistical analysis

Paired samples t-tests have been performed to quantify the blue component signal with respect to the total intensity signal (Red + Green + Blue) for each cell lines (Fig. 5). A type I error threshold of 0.05 was used for statistical significance.

### Single-cell classification and validation in mono-cultures

The computational classification was performed using Orange (Version 3.24.1) open-source software for data mining and ML-based on visual programming. Different cell lines were classified based on color-based features. The workflow of the visual algorithm is shown in Fig. S5. First, colorimetric features extracted using segmentation and feature selection algorithms were imported into Orange, and PCA was then performed to identify the top Principal Components (PC). The number of PCs was chosen to achieve 99% explained variance. Then, the transformed data are sent to the “test and score” widget where various models are tested, and their predictive scores are calculated based on a specific validation method. Specifically, the following ML models are used: (1) k-Nearest Neighbors (kNN, number of neighbors = 5, metric = Euclidean, and weight = uniform). (2) Logistic regression (regularization type = Lasso and strength = C1). (3) Random forest (number of trees = 10, the depth of the individual tree is limited to five, and subsets smaller than five are not split). (4) Support Vector Machine (SVM, cost = 1, regression loss = 0.1, kernel = linear, numerical tolerance = 0.001, and iteration limit = 100). (5) Neural network (neurons in hidden layers = 200, activation = ReLu, solver = Adam with regularization = 0.0001, and maximum number of iterations = 200). Model validation was performed using cross-validation (number of folds = 10, stratified). Results of the model validation are presented in tables showing the following scoring values: (1) accuracy = TP + TN/TP + FP + FN + TN, (2) precision = TP/TP + FP, (3) recall = TP/TP + FN, and (4) F1 score = 2 × (recall × precision)/(recall + precision), where TP = True Positives, TN = True Negatives, FP = False Positive, and FN = False Negatives. The size of the cell samples is N = 157 for MCF-10A, N = 152 for MDA-MB-231, N = 155 for MiaPaCa-2, and N = 58 for HUVECs. Further validation was performed by generating “*in silico* co-cultures” datasets by randomly combining the numeric values of the colorimetric features extracted from each cell line and using them as validation sets.

### Single-cell classification in co-cultures

The same method described in the previous paragraph was used for the case of co-cultures. Specifically, we co-cultured MDA-MB-231 with MCF-7 and MDA-MB-231 with MCF-10A. MDA-MB-231 was grown separately in their native media and labeled with Vybrant™ DiD cell-labeling solution (emission wavelength: 665 nm) (Invitrogen™, USA) before co-culture. This allowed us to distinguish each cell type in the co-culture via fluorescence imaging. Therefore, colored brightfield images and fluorescence images were acquired. Both sets of images were then segmented and a dataset was produced for each co-culture, containing the 16 colorimetric features together with their type, which was extracted from the fluorescence image.

## SUPPLEMENTARY MATERIAL

See the supplementary material for the supplementary figures and characterization of the effects of the ethanol treatment on cell viability and growth curves.

## AUTHORS' CONTRIBUTIONS

Y.B. conceived and led the project. V.L.M.V. and J.S.P. cultured the cells lines. Y.B., V.L.M.V., and J.S.P. performed the imaging. D.S.J. developed the automated computational single-cell segmentation and feature extraction algorithm. Y.B. conducted data analysis and computational classification. Y.B. wrote this manuscript. C.T.L. supervised this project. All the authors discussed the results and edited this manuscript. D.S.J., J.S.P., and V.L.M.V. contributed equally to this work.

## Data Availability

The data that support the findings of this study are available from the corresponding author upon reasonable request.

## References

[1] J. Icha , M. Weber , J. C. Waters , and C. Norden , BioEssays 39, 1700003–1700015 (2017). 10.1002/bies.201700003 28749075

[2] J. Y. Tinevez , J. Dragavon , L. Baba-Aissa , P. Roux , E. Perret , A. Canivet , V. Galy , and S. Shorte , Methods Enzymol. 506, 291–309 (2012). 10.1016/B978-0-12-391856-7.00039-1 22341230

[3] G. D. Johnson , R. S. Davidson , K. C. McNamee , G. Russell , D. Goodwin , and E. J. Holborow , J. Immunol. Methods 55, 231–242 (1982). 10.1016/0022-1759(82)90035-7 6819318

[4] J. Marrison , L. Räty , P. Marriott , and P. O'Toole , “Ptychography-a label free, high-contrast imaging technique for live cells using quantitative phase information,” Sci. Rep. 3, 1–7 (2013). 10.1038/srep02369 PMC3734479 23917865

[5] S. P. Denker and D. L. Barber , J. Cell Biol. 159, 1087–1096 (2002). 10.1083/jcb.200208050 12486114 PMC2173980

[6] C. Frantz , G. Barreiro , L. Dominguez , X. Chen , R. Eddy , J. Condeelis , M. J. S. Kelly , M. P. Jacobson , and D. L. Barber , J. Cell Biol. 183, 865–879 (2008). 10.1083/jcb.200804161 19029335 PMC2592832

[7] T. Sano , N. Kutsuna , D. Becker , R. Hedrich , and S. Hasezawa , Plant J. 57, 55–64 (2009). 10.1111/j.1365-313X.2008.03672.x 18778403

[8] D. Lagadic-Gossmann , L. Huc , and V. Lecureur , Cell Death Differ. 11, 953–961 (2004). 10.1038/sj.cdd.4401466 15195071

[9] S. Matsuyama , J. Llopis , Q. L. Deveraux , R. Y. Tsien , and J. C. Reed , Nat. Cell Biol. 2, 318–325 (2000). 10.1038/35014006 10854321

[10] R. A. Gottlieb , J. Nordberg , E. Skowronski , and B. M. Babior , Proc. Natl. Acad. Sci. U. S. A. 93, 654–658 (1996). 10.1073/pnas.93.2.654 8570610 PMC40107

[11] P. K. Hepler , Plant Physiol. 170, 3–22 (2016). 10.1104/pp.15.01506 26722019 PMC4704593

[12] S. Köhler , K. M. Schmoller , A. H. Crevenna , A. R. Bausch , E. Biophysik , and T. U. München , Cell Rep. 2, 433–439 (2012). 10.1016/j.celrep.2012.08.014 22999933 PMC3767110

[13] A. Asokan and M. J. Cho , J. Pharm. Sci. 91, 903–913 (2002). 10.1002/jps.10095 11948528

[14] E. Proksch , J. Dermatol. 45, 1044–1052 (2018). 10.1111/1346-8138.14489 29863755

[15] H. Prasad and R. Rao , Proc. Natl. Acad. Sci. U. S. A. 115, E6640–E6649 (2018). 10.1073/pnas.1801612115 29946028 PMC6048470

[16] Q. Wan , S. Chen , W. Shi , L. Li , and H. Ma , Angew. Chem., Int. Ed. 126, 11096–11100 (2014). 10.1002/ange.201405742

[17] A. Hulikova , A. L. Harris , R. D. Vaughan-Jones , and P. Swietach , J. Cell. Physiol. 228, 743–752 (2013). 10.1002/jcp.24221 22949268

[18] B. A. Webb , M. Chimenti , M. P. Jacobson , and D. L. Barber , Nat. Rev. Cancer 11, 671–677 (2011). 10.1038/nrc3110 21833026

[19] K. A. White , B. K. Grillo-Hill , and D. L. Barber , J. Cell Sci. 130, 663–669 (2017). 10.1242/jcs.195297 28202602 PMC5339414

[20] S. J. Reshkin , A. Bellizzi , S. Caldeira , V. Albarani , I. Malanchi , M. Poignee , M. Alunni‐Fabbroni , V. Casavola , and M. Tommasino , FASEB J. 14, 2185–2197 (2000). 10.1096/fj.00-0029com 11053239

[21] R. A. Cardone , V. Casavola , and S. J. Reshkin , Nat. Rev. Cancer 5, 786–795 (2005). 10.1038/nrc1713 16175178

[22] S. R. Amith , J. M. Wilkinson , and L. Fliegel , Biochem. Pharmacol. 118, 31–39 (2016). 10.1016/j.bcp.2016.08.010 27521504

[23] M. H. Lee , J. H. Han , J. H. Lee , N. Park , R. Kumar , C. Kang , and J. S. Kim , Angew. Chem., Int. Ed. 52, 6206–6209 (2013). 10.1002/anie.201301894 23610070

[24] W. Shi , X. Li , and H. Ma , Angew. Chem., Int. Ed. 51, 6432–6435 (2012). 10.1002/anie.201202533 22644672

[25] J. Tian , L. Ding , H. J. Xu , Z. Shen , H. Ju , L. Jia , L. Bao , and J. S. Yu , J. Am. Chem. Soc. 135, 18850–18858 (2013). 10.1021/ja408286k 24294991

[26] B. Tang , F. Yu , P. Li , L. Tong , X. Duan , T. Xie , and X. Wang , J. Am. Chem. Soc. 131, 3016–3023 (2009). 10.1021/ja809149g 19199620

[27] Y. Cao , R.-C. Qian , D.-W. Li , and Y.-T. Long , Chem. Commun. 51, 17584–17587 (2015). 10.1039/C5CC07697H 26477858

[28] F. Wang , R. G. Widejko , Z. Yang , K. T. Nguyen , H. Chen , L. P. Fernando , K. A. Christensen , and J. N. Anker , Anal. Chem. 84, 8013–8019 (2012). 10.1021/ac3018179 22881392

[29] H. Hou , Y. Zhao , C. Li , M. Wang , X. Xu , and Y. Jin , “Single-cell pH imaging and detection for pH profiling and label-free rapid identification of cancer-cells,” Sci. Rep. 7, 1–8 (2017). 10.1038/s41598-017-01956-1 28496209 PMC5431805

[30] M. Doan and A. E. Carpenter , Nat. Mater. 18, 414–418 (2019). 10.1038/s41563-019-0339-y 31000804

[31] E. Moen , D. Bannon , T. Kudo , W. Graf , M. Covert , and D. Van Valen , Nat. Methods 16, 1233–1246 (2019). 10.1038/s41592-019-0403-1 31133758 PMC8759575

[32] P. H. C. Chen , Y. Liu , and L. Peng , Nat. Mater. 18, 410–414 (2019). 10.1038/s41563-019-0345-0 31000806

[33] J. Candia , R. Maunu , M. Driscoll , A. Biancotto , P. Dagur , J. P. McCoy , H. N. Sen , L. Wei , A. Maritan , K. Cao , R. B. Nussenblatt , J. R. Banavar , and W. Losert , PLoS Comput. Biol. 9, e1003215 (2013). 10.1371/journal.pcbi.1003215 24039568 PMC3763994

[34] A. E. Carpenter , T. R. Jones , M. R. Lamprecht , C. Clarke , I. H. Kang , O. Friman , D. A. Guertin , J. H. Chang , R. A. Lindquist , J. Moffat , P. Golland , and D. M. Sabatini , Genome Biol. 7, R100 (2006). 10.1186/gb-2006-7-10-r100 17076895 PMC1794559

[35] S. Lee , T. V. Axelsen , A. P. Andersen , P. Vahl , S. F. Pedersen , and E. Boedtkjer , Oncogene 35, 2112–2122 (2016). 10.1038/onc.2015.273 26212013

[36] D. Cong , W. Zhu , Y. Shi , K. B. Pointer , P. A. Clark , H. Shen , J. S. Kuo , S. Hu , and D. Sun , Carcinogenesis 35, 2014–2024 (2014). 10.1093/carcin/bgu089 24717311 PMC4146414

[37] L. Counillon , Y. Bouret , I. Marchiq , and J. Pouysségur , Biochim. Biophys. Acta-Mol. Cell Res. 1863, 2465–2480 (2016). 10.1016/j.bbamcr.2016.02.018 26944480

[38] F. A. Gallagher , H. Sladen , M. I. Kettunen , E. M. Serrao , T. B. Rodrigues , A. Wright , A. B. Gill , S. McGuire , T. C. Booth , J. Boren , A. McIntyre , J. L. Miller , S. H. Lee , D. Honess , S. E. Day , D. E. Hu , W. J. Howat , A. L. Harris , and K. M. Brindle , Cancer Res. 75, 4109–4118 (2015). 10.1158/0008-5472.CAN-15-0857 26249175 PMC4594768

[39] Y. Shen , L. Liang , S. Zhang , D. Huang , J. Zhang , S. Xu , C. Liang , and W. Xu , Nanoscale 10, 1622–1630 (2018). 10.1039/C7NR08636A 29239454

[40] P. Marquet , B. Rappaz , P. J. Magistretti , E. Cuche , Y. Emery , T. Colomb , and C. Depeursinge , Opt. Lett. 30, 468–470 (2005). 10.1364/OL.30.000468 15789705

[41] N. Pavillon , J. Kühn , C. Moratal , P. Jourdain , C. Depeursinge , P. J. Magistretti , and P. Marquet , PLoS One 7(1), e30912 (2012). 10.1371/journal.pone.0030912 22303471 PMC3269420

[42] Y. Cotte , F. Toy , P. Jourdain , N. Pavillon , D. Boss , P. Magistretti , P. Marquet , and C. Depeursinge , Nat. Photonics 7, 113–117 (2013). 10.1038/nphoton.2012.329

[43] M. Beleggia , M. A. Schofield , V. V. Volkov , and Y. Zhu , Ultramicroscopy 102, 37–49 (2004). 10.1016/j.ultramic.2004.08.004 15556699

[44] A. M. Maiden , J. M. Rodenburg , and M. J. Humphry , “A new method of high resolution, quantitative phase scanning microscopy,” Scanning Microsc. 7729, 77291I (2010). 10.1117/12.853339

[45] J. M. Rodenburg , A. C. Hurst , and A. G. Cullis , Ultramicroscopy 107, 227–231 (2007). 10.1016/j.ultramic.2006.07.007 16959428

[46] J. S. Dudani , D. R. Gossett , H. T. K. Tse , and D. Di Carlo , Lab Chip 13, 3728–3734 (2013). 10.1039/c3lc50649e 23884381

[47] O. Otto , P. Rosendahl , A. Mietke , S. Golfier , C. Herold , D. Klaue , S. Girardo , S. Pagliara , A. Ekpenyong , A. Jacobi , M. Wobus , N. Töpfner , U. F. Keyser , J. Mansfeld , E. Fischer-Friedrich , and J. Guck , Nat. Methods 12, 199–202 (2015). 10.1038/nmeth.3281 25643151

[48] Y. Belotti , S. Tolomeo , M. J. Conneely , T. Huang , S. J. McKenna , G. Nabi , and D. McGloin , Sci. Rep. 9, 1–9 (2019). 10.1038/s41598-019-42008-0 30952895 PMC6450875

[49] P. Rosendahl , K. Plak , A. Jacobi , M. Kraeter , N. Toepfner , O. Otto , C. Herold , M. Winzi , M. Herbig , Y. Ge , S. Girardo , K. Wagner , B. Baum , and J. Guck , Nat. Methods 15, 355–358 (2018). 10.1038/nmeth.4639 29608556

[50] J. R. Casey , S. Grinstein , and J. Orlowski , Nat. Rev. Mol. Cell Biol. 11, 50–61 (2010). 10.1038/nrm2820 19997129

[51] J. Michl , K. C. Park , and P. Swietach , Commun. Biol. 2, 1–12 (2019). 10.1038/s42003-019-0393-7 31044169 PMC6486606

[52] L. Van der Maaten and G. Hinton , “Visualizing data using t-SNE,” J. Mach. Learn. Res. 9, 2579–2605 (2008).

